# Percutaneous transvenous mitral commissurotomy during pregnancy: a contemporary Southeast Asian experience

**DOI:** 10.1186/s12884-026-09824-5

**Published:** 2026-08-15

**Authors:** Suchat Sricholwattana, Paisit Kosum, Nonthikorn Theerasuwipakorn, Suphot Srimahachota

**Affiliations:** 1 Division of Cardiovascular Medicine, Department of Medicine, Faculty of Medicine, Srinakharinwirot University, Nakhon Nayok, Thailand; 2 Cardiac Center, King Chulalongkorn Memorial Hospital, Bangkok, Thailand; 3 Division of Cardiovascular Medicine, Department of Medicine, Faculty of Medicine, Naresuan University, Phitsanulok, 65000 Thailand; 4 Cardiac Center, Naresuan University Hospital, Phitsanulok, Thailand; 5 Division of Cardiovascular Medicine, Department of Medicine, Faculty of Medicine, Chulalongkorn University, King Chulalongkorn Memorial Hospital, Bangkok, Thailand

**Keywords:** Rheumatic heart disease, Mitral stenosis, Pregnancy, Percutaneous transvenous mitral commissurotomy, Maternal outcomes, Fetal outcomes

## Abstract

**Background:**

Severe rheumatic mitral stenosis during pregnancy is associated with substantial maternal and fetal morbidity because physiological hemodynamic changes may precipitate heart failure and pulmonary hypertension. Percutaneous transvenous mitral commissurotomy (PTMC) is the recommended intervention for carefully selected symptomatic patients who remain refractory to medical therapy. Although PTMC during pregnancy has been widely reported, contemporary data describing procedural characteristics, radiation exposure, and maternal–fetal outcomes from Southeast Asia remain limited. This study aimed to evaluate the procedural, hemodynamic, radiation, maternal, and neonatal outcomes of PTMC performed during pregnancy.

**Methods:**

We conducted a retrospective observational study of consecutive pregnant women with rheumatic mitral stenosis who underwent PTMC at a tertiary referral center in Thailand between 2002 and 2022 according to the institutional eligibility criteria used during the study period. Given the limited sample size, all analyses were considered exploratory. Hemodynamic, echocardiographic, procedural, radiation, maternal, and neonatal outcomes were evaluated.

**Results:**

Eighteen pregnant women underwent PTMC at a mean gestational age of 24.6 ± 5.7 weeks. Procedural success was achieved in 94% of patients. Mitral valve area significantly increased from 0.98 ± 0.23 cm² to 1.50 ± 0.34 cm² (*p* < 0.001), while mean transmitral gradient decreased from 15.98 ± 5.97 mmHg to 7.26 ± 3.92 mmHg (*p* = 0.001). Mean left atrial pressure also significantly decreased from 31.94 ± 5.95 mmHg to 17.41 ± 4.29 mmHg (*p* < 0.001), and most patients improved from New York Heart Association functional class III–IV to class I–II (*p* < 0.001). Among the 12 patients with available radiation data, the median fluoroscopy time was 3.76 min and the median cumulative air Kerma was 21 mGy. Among patients with available obstetric follow-up (*n* = 12), no maternal deaths, neonatal deaths, or congenital anomalies were observed.

**Conclusions:**

In this small retrospective single-center study, PTMC was associated with significant immediate hemodynamic and symptomatic improvement in carefully selected pregnant women with rheumatic mitral stenosis. Among patients with available follow-up, maternal and neonatal outcomes were generally favorable; however, these findings should be interpreted cautiously because of the limited sample size, prolonged recruitment period, retrospective design, and incomplete follow-up. Larger prospective multicenter studies are required to confirm these findings.

## Introduction

Rheumatic heart disease (RHD) remains the leading cause of valvular heart disease during pregnancy in low- and middle-income countries, including Thailand [1, 2]. Among valvular lesions, mitral stenosis is the most frequently encountered and represents a substantial proportion of cardiovascular disease in pregnancy in developing regions [3, 4]. The hemodynamic adaptations of pregnancy, including increased plasma volume, heart rate, and cardiac output, markedly increase transmitral flow and left atrial pressure, thereby exacerbating the severity of mitral stenosis. Consequently, pregnant women with significant mitral stenosis are at increased risk of heart failure, pulmonary hypertension, atrial arrhythmias, and adverse maternal and fetal outcomes [5].

According to the 2025 European Society of Cardiology (ESC) Guidelines for the management of cardiovascular diseases during pregnancy, severe mitral stenosis is classified as a high-risk cardiac condition. Intervention prior to pregnancy is recommended in women with a mitral valve area < 1.5 cm². During pregnancy, medical therapy including activity restriction, beta-blockers, and diuretics may provide symptomatic relief; however, percutaneous mitral commissurotomy should be considered in patients with persistent severe symptoms or systolic pulmonary artery pressure > 50 mmHg despite optimal medical therapy [3]. Compared with surgical commissurotomy, percutaneous transvenous mitral commissurotomy (PTMC) offers a less invasive approach and has become the preferred intervention in appropriately selected pregnant patients [6, 7].

Although PTMC during pregnancy has been extensively investigated over the past three decades, most published studies originated from South Asia or the Middle East, whereas contemporary data from Southeast Asia remain limited. Few reports have comprehensively described procedural radiation indices together with maternal and neonatal outcomes in routine clinical practice. Therefore, this study aimed to describe the contemporary experience of PTMC performed during pregnancy at a tertiary referral center in Thailand, with particular emphasis on procedural characteristics, radiation exposure, hemodynamic improvement, and available maternal and neonatal outcomes.

## Methods

### Study design and population

This retrospective observational study included consecutive pregnant women with rheumatic mitral stenosis who underwent PTMC at King Chulalongkorn Memorial Hospital, Bangkok, Thailand, between January 2002 and December 2022. All eligible patients were identified from institutional procedural and echocardiographic databases.

Eligible patients were pregnant women in the second or third trimester with rheumatic mitral stenosis who met the institutional criteria for PTMC during the study period. Patients were considered eligible if they had a mitral valve area (MVA) ≤ 1.0 cm² or a mean transmitral pressure gradient ≥ 10 mmHg, together with persistent symptoms despite optimal medical therapy or acute pulmonary edema during the index pregnancy. Patients were required to have favorable valve morphology based on the Wilkins score [8]. The decision to perform PTMC was made by a multidisciplinary heart team based on the overall clinical presentation, valve morphology, and procedural suitability.

### Echocardiographic assessment

All patients underwent comprehensive transthoracic echocardiography prior to PTMC, including two-dimensional imaging, Doppler assessment, and color flow mapping. Mitral valve morphology was evaluated using the Wilkins score [8], including leaflet mobility, thickness, calcification, and subvalvular involvement.

Key echocardiographic parameters included mitral valve area, peak and mean transmitral gradient, and systolic pulmonary artery pressure. Post-procedural echocardiography was performed within 24 h after PTMC to assess procedural outcomes and complications, including mitral regurgitation severity.

### PTMC procedure

PTMC was performed using a transseptal antegrade approach with a single Inoue balloon technique by experienced interventional cardiologists. All procedures were performed under local anesthesia with transesophageal echocardiographic guidance. Abdominal lead shielding was routinely applied, and fluoroscopy was performed using a low-dose protocol (7.5 frames/s) following the ALARA principle. Routine cineangiography was not performed because procedural guidance relied primarily on fluoroscopy in conjunction with transesophageal echocardiography. Procedural data collected included total procedure time, fluoroscopy time, cumulative air Kerma (mGy), and kerma-area product (Gy·cm²).

Procedural success was defined as a final mitral valve area ≥ 1.5 cm² or an increase in mitral valve area ≥ 50% from baseline without severe mitral regurgitation.

### Outcomes

The primary outcome was procedural success. Secondary outcomes included changes in mitral valve area, transmitral gradient, left atrial pressure, pulmonary artery pressure, NYHA functional class, procedural characteristics, radiation exposure, maternal outcomes (maternal survival and need for repeat intervention), obstetric outcomes (gestational age at delivery, mode of delivery, complications), and neonatal outcomes (birth weight, Apgar score, and complications).

Preterm birth was defined as delivery before 37 weeks of gestation, and intrauterine growth restriction (IUGR) was defined according to standard obstetric criteria.

Follow-up data were collected through delivery and available postpartum clinical records. Because King Chulalongkorn Memorial Hospital is a national referral center, many patients returned to their local hospitals after PTMC for antenatal care and delivery; therefore, complete obstetric and neonatal follow-up was unavailable for all patients.

### Ethical approval

This study was approved by the Institutional Review Board of the Faculty of Medicine, Chulalongkorn University, Bangkok, Thailand (COA No. 0678/2023; IRB No. 0022/66). The study was conducted in accordance with the Declaration of Helsinki. Due to the retrospective nature of the study, the requirement for informed consent was waived. Patient data were anonymized prior to analysis.

### Statistical analysis

Continuous variables are presented as mean ± standard deviation or median (interquartile range), depending on data distribution. Categorical variables are expressed as frequencies and percentages.

Data distribution was assessed by visual inspection and the Kolmogorov–Smirnov test. Continuous variables were analyzed using paired t-tests or Wilcoxon signed-rank tests as appropriate. Changes in NYHA functional class were evaluated using the Stuart–Maxwell test for marginal homogeneity.

All statistical analyses were performed using IBM SPSS Statistics version 29.0 (IBM Corp., Armonk, NY, USA). A two-sided p-value < 0.05 was considered statistically significant. Given the limited sample size, all analyses were considered exploratory. Complete-case analysis was performed for variables with missing follow-up data, and no imputation was undertaken.

## Results

### Patient characteristics

A total of 18 consecutive pregnant women with rheumatic mitral stenosis underwent PTMC during the study period. The mean maternal age was 29.39 ± 4.72 years, and the mean gestational age at intervention was 24.56 ± 5.70 weeks. Twelve patients (66.7%) underwent PTMC during the second trimester, whereas six (33.3%) underwent the procedure during the third trimester.

Seven women (38.9%) were primigravida. Hypertension and diabetes mellitus were present in one (5.6%) and two (11.1%) patients, respectively. Three patients (16.7%) presented with acute pulmonary edema before intervention and underwent urgent PTMC, whereas the remaining patients underwent elective procedures after optimization of medical therapy.

At baseline, four patients (22.2%) were in NYHA functional class II, eleven (61.1%) were in class III, and three (16.7%) were in class IV. The mean left ventricular ejection fraction was 61.38 ± 8.54%, indicating preserved systolic function. The mean Wilkins score was 7.89 ± 1.49, with favorable valve morphology (Wilkins score ≤ 8) observed in 13 patients (72.2%). Baseline patient characteristics are summarized in Table 1.

**Table 1:** Baseline characteristics of pregnant women undergoing PTMC (*n* = 18)

Variable		Value
Age (years)		29.39 ± 4.72
Gestational age at PTMC (weeks)		24.56 ± 5.70
Second trimester		12 (66.7%)
Third trimester		6 (33.3%)
Primigravida		7 (38.9%)
Body weight (kg)		56.98 ± 7.79
Height (cm)		154 ± 5.7
Hypertension		1 (5.6%)
Diabetes mellitus		
Gestational diabetes Pregestational diabetes		2 (11.1%) 1 (5.6%)
Acute pulmonary edema		3 (16.7%)
NYHA functional class	II	4 (22.2%)
	III	11 (61.1%)
	IV	3 (16.7%)
Left ventricular ejection fraction (%)		61.38 ± 8.54
Wilkins score		7.89 ± 1.49
≤ 8 > 8		13 (72.2%) 5 (27.8%)

*Abbreviations*: *PTMC* percutaneous transvenous mitral commissurotomy, *NYHA* New York Heart Association

### Procedural characteristics

Technically, PTMC was successfully completed in all 18 patients using the Inoue balloon technique. Procedural success, defined as achievement of the predefined echocardiographic criteria without severe mitral regurgitation, was achieved in 17 patients (94%). All procedures were performed under local anesthesia with transesophageal echocardiographic guidance. Abdominal lead shielding was routinely applied throughout the procedure, and fluoroscopy was performed using a low-dose protocol to minimize fetal radiation exposure.

The mean procedure time was 38.56 ± 9.10 min. Radiation data were available for 12 patients. Among these patients, the median fluoroscopy time was 3.76 min (interquartile range [IQR] 2.10–4.50), the median cumulative air Kerma was 21.0 mGy (IQR 12.92–30.23), and the median kerma-area product was 3.080 Gy·cm² (IQR 2.664–3.862).

Eight patients (44.4%) required a single balloon inflation, whereas the remaining patients required two or more inflations to achieve satisfactory commissural splitting. Detailed procedural characteristics are presented in Table 2. No procedure-related mortality, cardiac tamponade, stroke, or emergency cardiac surgery occurred during hospitalization.

**Table 2:** Procedural characteristics of PTMC (*n* = 18)

Variable		Value
First PTMC		15 (83.3%)
Procedure time (min)		38.56 ± 9.1
Fluoroscopy time (min) (*n* = 12)		3.76 (2.1–4.5)
Kerma-area product (Gy·cm²) (*n* = 12)		3.080 (2.664–3.862)
Cumulative air Kerma (mGy) (*n* = 12)		21 (12.92–30.23)
Inoue balloon technique		18 (100%)
Number of dilatations	1	8 (44.4%)
	2	3 (16.7%)
	3	5 (27.8%)
	4	1 (5.6%)
	5	1 (5.6%)
Maximum balloon size		25.44 ± 1.62

*Abbreviations*: *PTMC* percutaneous transvenous mitral commissurotomy

### Hemodynamic and echocardiographic outcomes

PTMC resulted in significant improvements in hemodynamic and echocardiographic parameters (Table 3).

**Table 3:** Hemodynamic and echocardiographic outcomes before and after PTMC

Parameter	Pre-PTMC	Post-PTMC	Mean difference (95% CI)	*p*-value
Left atrial pressure (mmHg)	31.94 ± 5.95	17.41 ± 4.29	-14.12 (-17.01, -11.23)	< 0.001
Mitral valve area (cm²)	0.98 ± 0.23	1.50 ± 0.34	0.48 (0.28, 0.67)	< 0.001
Mean transmitral gradient (mmHg)				
On catheterization	19.78 ± 5.22	8.18 ± 4.3	-11.59 (-13.75, -9.43)	< 0.001
By echocardiography	15.98 ± 5.97	7.26 ± 3.92	−8.26 (− 12.45, − 4.07)	0.001
Peak transmitral gradient (mmHg)^a^	30.5 ± 8.96	10.65 ± 3.86	-22.6 (-39.69, -5.51)	0.024
Pulmonary artery pressure (PAP) (mmHg)^a^				
Systolic PAP	64.33 ± 26.13	48.47 ± 22.23	-14 (-25.89, -2.11)	0.024
Diastolic PAP	29.13 ± 17.07	17.07 ± 6.72	-12.5 (-32.61, 7.61)	0.171
Mean PAP	38 ± 23.69	27.79 ± 11.85	-16.33 (-35.14, 2.47)	0.076

Mitral regurgitation				
Severity	Pre-PTMC		Post-PTMC	
None	11 (61.1%)		1 (5.6%)	
Mild	7 (38.9%)		15 (83.3%)	
Moderate	0		1 (5.6%)	
Severe	0		1 (5.6%)	

Comparisons between pre- and post-procedural parameters were performed using paired t-tests or Wilcoxon signed-rank tests, as appropriate

*Abbreviations*: *PTMC* percutaneous transvenous mitral commissurotomy, *PAP* Pulmonary artery pressure

^a^ By Echocardiography

Mitral valve area increased significantly from 0.98 ± 0.23 cm² before PTMC to 1.50 ± 0.34 cm² after the procedure (mean difference 0.48 cm², 95% CI 0.28–0.67; *p* < 0.001). Mean left atrial pressure decreased from 31.94 ± 5.95 mmHg to 17.41 ± 4.29 mmHg (mean difference − 14.12 mmHg, 95% CI − 17.01 to − 11.23; *p* < 0.001). The mean transmitral pressure gradient measured during cardiac catheterization decreased significantly from 19.78 ± 5.22 mmHg to 8.18 ± 4.30 mmHg (mean difference − 11.59 mmHg, 95% CI − 13.75 to − 9.43; *p* < 0.001). Similarly, the echocardiographic mean transmitral gradient decreased from 15.98 ± 5.97 mmHg to 7.26 ± 3.92 mmHg (mean difference − 8.26 mmHg, 95% CI − 12.45 to − 4.07; *p* = 0.001). Peak transmitral gradient measured by echocardiography also decreased significantly from 30.50 ± 8.96 mmHg to 10.65 ± 3.86 mmHg (mean difference − 22.60 mmHg, 95% CI − 39.69 to − 5.51; *p* = 0.024). Systolic pulmonary artery pressure decreased from 64.33 ± 26.13 mmHg to 48.47 ± 22.23 mmHg (mean difference − 14.00 mmHg, 95% CI − 25.89 to − 2.11; *p* = 0.024). Although reductions in diastolic and mean pulmonary artery pressures were observed, these changes did not reach statistical significance.

Mitral regurgitation severity increased after PTMC. Before intervention, 11 patients (61.1%) had no mitral regurgitation and seven (38.9%) had mild regurgitation. Following PTMC, mild mitral regurgitation was observed in 15 patients (83.3%), whereas moderate and severe mitral regurgitation each occurred in one patient (5.6%). The single patient with severe mitral regurgitation represented the only procedural failure according to the predefined study criteria.

### Functional outcomes

**Fig. 1:**
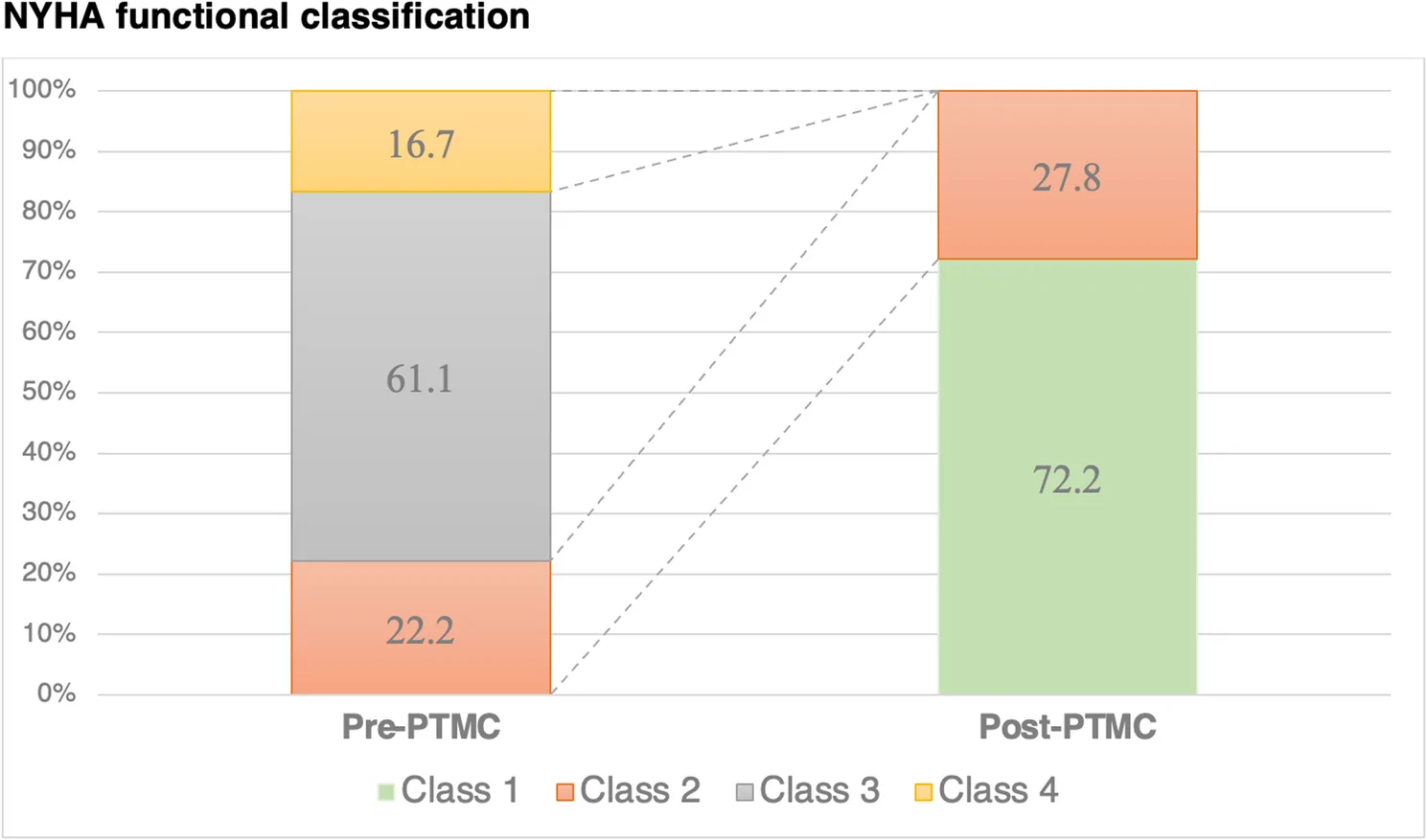
Change in New York Heart Association (NYHA) functional class before and after percutaneous transvenous mitral commissurotomy (PTMC). Paired comparison demonstrating significant improvement in NYHA functional class following PTMC (Stuart–Maxwell test for marginal homogeneity, *p* < 0.001)

NYHA functional class improved significantly following PTMC (Fig. 1). Before the procedure, 14 patients (77.8%) were in NYHA functional class III–IV, whereas four patients (22.2%) were in class II. Following PTMC, all patients improved to NYHA functional class I–II, with 13 patients (72.2%) achieving class I and the remaining five patients (27.8%) classified as class II. The overall improvement in functional class was statistically significant (*p* < 0.001).

### Maternal and obstetric outcomes

Maternal and obstetric follow-up data were available for 12 patients (66.7%). All women survived to delivery, and no patient required repeat PTMC or mitral valve surgery during the available follow-up period.

The mean gestational age at delivery was 36.55 ± 2.62 weeks. Vaginal delivery was achieved in eight patients (66.7%), whereas four patients (33.3%) underwent cesarean delivery. Preterm birth occurred in two patients (16.7%), and intrauterine growth restriction was documented in one patient (8.3%). Maternal and obstetric outcomes are summarized in Table 4. Because this was a tertiary referral center, several patients returned to their local hospitals for antenatal care and delivery after PTMC; therefore, complete obstetric follow-up was unavailable for all patients.

**Table 4:** Maternal and neonatal outcomes (*n* = 12)

Variable	Value
Gestational age at delivery (weeks)	36.55 ± 2.62
Mode of delivery	
Vaginal delivery	8 (66.7%)
Cesarean section	4 (33.3%)
Apgar score at 1 min	7.6 ± 2.63
Apgar score at 5 min	9.1 ± 1.73
Preterm birth	2 (16.7%)
IUGR	1 (8.3%)
Birth weight (kg)	2.48 ± 0.62
Low birth weight (≤ 2.5 kg)	3 (25%)
Neonatal death	0
Congenital anomalies	0
Maternal death	0

Available outcome data varied across variables because of incomplete retrospective follow-up and missing obstetric records

*Abbreviations*: *PTMC* percutaneous transvenous mitral commissurotomy, *IUGR* Intrauterine growth restriction

### Neonatal outcomes

Neonatal outcome data were available for the same 12 pregnancies with documented delivery records. The mean birth weight was 2.48 ± 0.62 kg, and three neonates (25.0%) had a birth weight ≤ 2.5 kg. The mean Apgar scores at 1 and 5 min were 7.6 ± 2.63 and 9.1 ± 1.73, respectively. No neonatal deaths or congenital anomalies were identified among patients with available follow-up. Detailed neonatal outcomes are presented in Table 4.

**Fig. 2:**
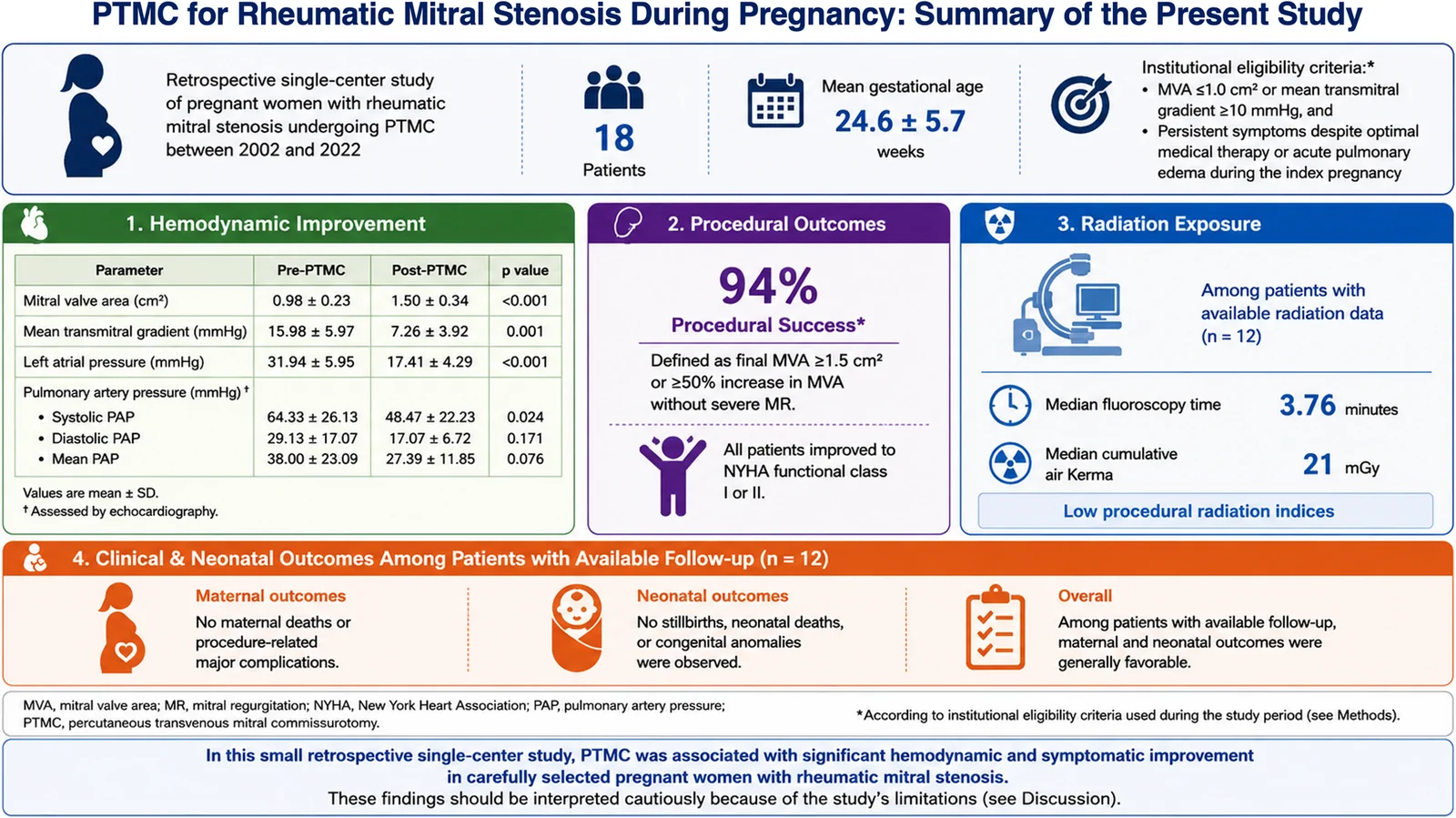
Central Illustration. Graphical summary of the principal findings of the present study. The illustration summarizes the study population, institutional eligibility criteria applied during the study period, procedural outcomes, hemodynamic improvements, procedural radiation indices, and maternal and neonatal outcomes among patients with available follow-up

The principal findings of the present study are summarized graphically in Fig. 2.

## Discussion

Rheumatic mitral stenosis remains an important cause of cardiovascular morbidity during pregnancy in low- and middle-income countries where rheumatic heart disease continues to be prevalent [9–11]. Physiological increases in plasma volume, heart rate, and cardiac output during pregnancy substantially increase transmitral flow and left atrial pressure, thereby predisposing women with severe mitral stenosis to pulmonary edema, pulmonary hypertension, atrial arrhythmias, and heart failure [3–5, 12]. Current ESC guidelines recommend percutaneous mitral commissurotomy for carefully selected symptomatic patients with favorable valve morphology who remain symptomatic despite optimal medical therapy because it avoids cardiopulmonary bypass while providing effective relief of valvular obstruction [3, 6, 7].

In the present study, PTMC achieved the predefined procedural success criteria in 94% of patients and was associated with significant improvements in mitral valve area, transmitral pressure gradients, left atrial pressure, systolic pulmonary artery pressure, and NYHA functional class. One patient developed severe mitral regurgitation and therefore did not meet the predefined definition of procedural success. Overall, the magnitude of hemodynamic improvement observed in our study population is consistent with previous observational studies reporting procedural success rates between approximately 80% and 100% in appropriately selected pregnant women with rheumatic mitral stenosis [5, 6, 13–25].

The favorable procedural results observed in this study population may partly be explained by the predominance of favorable valve morphology. Previous studies have consistently demonstrated that lower Wilkins scores are associated with more effective commissural splitting, greater post-procedural mitral valve area, and a lower likelihood of severe mitral regurgitation following balloon commissurotomy [8].

An additional contribution of this study is the reporting of contemporary procedural and radiation parameters from a tertiary referral center in Southeast Asia. Among the 12 patients with available radiation measurements, fluoroscopy time and cumulative air Kerma were low. It should be emphasized that cumulative air Kerma and kerma-area product are procedural radiation indices and should not be interpreted as direct estimates of fetal radiation dose because fetal radiation exposure was not directly measured. However, because radiation data were unavailable for one-third of the study population and the overall sample size was limited, these findings should be interpreted cautiously. Nevertheless, these findings suggest that PTMC may be performed using radiation-minimization strategies—including transesophageal echocardiographic guidance, abdominal lead shielding, and low-dose fluoroscopy— in experienced centers.

Maternal and neonatal outcome data were available for 12 patients. Within this subgroup, no maternal deaths, neonatal deaths, congenital anomalies, repeat PTMC, or mitral valve surgery were observed during the available follow-up period. However, these findings should be interpreted descriptively rather than as definitive evidence of procedural safety because outcome data were incomplete and follow-up duration varied among patients. The referral nature of our institution resulted in many patients returning to their local hospitals after PTMC, limiting complete longitudinal follow-up.

The findings of the present study should also be interpreted alongside previously published evidence summarized in Table 5. Across multiple observational studies, PTMC has consistently been associated with immediate hemodynamic improvement in appropriately selected pregnant women with severe rheumatic mitral stenosis. Our results are broadly consistent with these reports while providing additional contemporary procedural information from Thailand, including radiation parameters that have been infrequently reported in previous studies [16, 20, 22–25].

**Table 5:** Summary of published studies evaluating PTMC during pregnancy in severe rheumatic mitral stenosis

Study (Year)	*N*	GA (weeks)	NYHA class	Success (%)	Fluoroscopy time / Radiation exposure	MVA pre ◊ post (cm^2^)	Gradient / PAP change	Maternal complications	Fetal outcomes
Kalra et al., 1994	27	22 ± 4	III–IV	96	NR	0.78 → 2.2	Gradient 30.5 → 6.1 mmHg	1 severe MR (surgery)	Mostly term, favorable
Ben Farhat et al., 1997	44	NR	Severe MS	100	NR	0.96 → 2.4	Gradient 22 → 5 mmHg	Minimal	Favorable
Nercolini et al., 2002	44	23 ± 6	Symptomatic	95	NR	1.17 → 2.06	Gradient 16.2 → 7.9 mmHg	No major complications	81% term, some prematurity
de Souza et al., 2001	21	NR	Severe HF	95	NR	Significant ↑	Significant ↓	Lower vs. surgery	Lower fetal mortality
Vinayakumar et al., 2016	49	23.5 ± 5.2	II–IV	95.9	NR	0.93 → 1.75	PAP 43 → 22 mmHg	Tamponade (*n* = 1), MR (*n* = 1)	81% term, 1 abortion
Pillai et al., 2018	96	23.4 ± 10.9	Severe HF	80.2	NR	NR	Sustained ↓	Severe MR 5.2%, mortality in advanced HF	Improved if successful
Sharma et al., 2018	10	NR	Severe MS	High	NR	NR	NR	Clinical improvement	Comparable
JOMIVA study, 2017	NR	NR	Severe MS	High	NR	NR	Significant ↓	Safe	Favorable
ROPAC registry, 2018	390 (MS 273)	NR	Mixed	NR	NR	NR	NR	HF up to 49% in severe MS	Improved with intervention
Present Study	18	24.6 ± 5.7	III-IV	94	3.76 min / 21 mGy	0.98 → 1.50	Significant ↓	• No maternal death among available follow-up • 1 Severe MR • No repeat PTMC/MV surgery	• No neonatal death or congenital anomaly among available follow-up

*Abbreviations*: *MVA* mitral valve area, *NYHA *New York Heart Association functional class, *PTMC* percutaneous transvenous mitral commissurotomy, *BMV* balloon mitral valvuloplasty, *PMV* percutaneous mitral valvuloplasty, *MR* mitral regurgitation, *PAP *pulmonary artery pressure, *GA* gestational age, *HF* heart failure, *IUGR* intrauterine growth restriction, *MVC* mitral valve commissurotomy (surgical), *NR* not reported

The present findings are also supported by previously published long-term data from our institution demonstrating sustained hemodynamic improvement following PTMC in patients with rheumatic mitral stenosis [26]. Although the present study was not designed to evaluate long-term maternal or neonatal outcomes, the immediate procedural and hemodynamic findings are consistent with the established effectiveness of PTMC in appropriately selected patients.

Taken together, these findings are consistent with the continued role of PTMC as the preferred catheter-based intervention for carefully selected pregnant women with severe rheumatic mitral stenosis when performed by experienced multidisciplinary heart teams. Confirmation of maternal, fetal, and long-term clinical outcomes will require larger prospective multicenter studies with more complete follow-up.

Contemporary valvular heart disease guidelines generally define severe mitral stenosis as a mitral valve area ≤ 1.5 cm². However, because patient recruitment spanned two decades (2002–2022), patient selection in this retrospective study reflected the institutional eligibility criteria applied during the study period. These criteria incorporated both anatomical severity (mitral valve area ≤ 1.0 cm²) and hemodynamic severity (mean transmitral pressure gradient ≥ 10 mmHg) when determining suitability for PTMC. Therefore, they should be interpreted as historical institutional selection criteria rather than as a proposed contemporary definition of severe mitral stenosis. This also explains why a small number of patients with a mitral valve area > 1.0 cm² were included, as these patients met the institutional hemodynamic eligibility criterion of a mean transmitral pressure gradient ≥ 10 mmHg.

### Limitations

Several limitations of this study should be acknowledged. First, this was a retrospective single-center study with a relatively small sample size, limiting statistical power and the generalizability of the findings. Second, patient recruitment spanned approximately two decades (2002–2022), during which procedural techniques, imaging technology, operator experience, radiation-reduction strategies, and multidisciplinary cardio-obstetric care may have evolved. Third, maternal and neonatal follow-up data were unavailable for one-third of the study population because several patients were referred from other hospitals and returned to their local institutions for ongoing obstetric care after successful PTMC. Fourth, radiation parameters, including cumulative air Kerma and kerma-area product, were available for only a subset of patients, and fetal radiation exposure was not directly measured or estimated. Finally, the absence of a comparison group precludes direct assessment of the relative effectiveness of PTMC compared with alternative management strategies. Accordingly, the findings of the present study should be considered exploratory and hypothesis-generating rather than definitive evidence regarding the effectiveness or safety of PTMC during pregnancy. Larger prospective multicenter studies with standardized maternal and neonatal follow-up are warranted to validate these findings.

## Conclusion

In this retrospective single-center study, PTMC was associated with significant immediate hemodynamic and symptomatic improvement in carefully selected pregnant women with rheumatic mitral stenosis. Among patients with available follow-up, maternal and neonatal outcomes were generally favorable. However, because of the limited sample size, prolonged recruitment period, retrospective design, absence of a comparison group, and incomplete follow-up, these findings should be interpreted cautiously and considered exploratory and hypothesis-generating rather than definitive evidence regarding the effectiveness or safety of PTMC during pregnancy. Larger prospective multicenter studies with standardized maternal and neonatal follow-up are required to confirm these findings.

## Data Availability

The datasets generated and/or analyzed during the current study are not publicly available because they contain potentially identifiable patient information but are available from the corresponding author on reasonable request and with permission from the institutional review board.

## Abbreviations

PTMC: Percutaneous transvenous mitral commissurotomy
RHD: Rheumatic heart disease
MVA: Mitral valve area
NYHA: New York heart association
PAP: Pulmonary artery pressure
IUGR: Intrauterine growth restriction
ESC: European society of cardiology
